# The multidimensional prognostic index (MPI) predicts long-term mortality in old type 2 diabetes mellitus patients: a 13-year follow-up study

**DOI:** 10.1007/s40618-023-02135-y

**Published:** 2023-06-18

**Authors:** F. Salis, E. Cossu, A. Mandas

**Affiliations:** 1 Department of Medical Sciences, and Public Health, University of Cagliari, SS 554 bivio Sestu, 09042 Monserrato, Cagliari Italy; 2 University Hospital “Azienda Ospedaliero-Universitaria” of Cagliari, Cagliari, Italy

**Keywords:** Comprehensive geriatric assessment (CGA), Mortality, Elderly, Diabetes mellitus, Cardiovascular, Kidney

## Abstract

**Purpose:**

The Multidimensional Prognostic Index (MPI) is a tool capable of holistically frame older patients in different settings and affected by different pathologies, establishing a risk of adverse events. Among them, type 2 diabetes mellitus (T2DM), a common metabolic disease in the elderly, is responsible for complications and deaths. Few previous works have focused specifically on MPI and DM, and none have followed up the patients for more than 3 years. The aim of the present study is to analyze MPI accuracy in predicting mortality in a cohort of T2DM patients followed-up for 13 years.

**Methods:**

The enrolled subjects were evaluated with MPI, identifying three levels of risk: MPI1 (low risk, 0.0–0.33), MPI2 (moderate risk, 0.34–0.66), and MPI3 (severe risk, 0.67–1.0), and with glycated hemoglobin, and years since T2DM diagnosis.

**Results:**

One hundred and seven patients met the inclusion criteria. MPI3 was excluded by further analysis since it was made up of only three patients. Overall, cognitive performances, autonomies in daily living, nutritional status, risk of pressure injuries, comorbidities, and taken drugs were better (*p* ≤ 0.0077) in MPI1 than MPI2; moreover, the story of T2DM was shorter (*p* = 0.0026). Cox model showed an overall 13-year survival of 51.9%, and survival rates were significantly smaller in MPI2 (HR: 4.71, *p* = 0.0007). Finally, increased age (HR: 1.15), poorer cognitive abilities (HR: 1.26), vascular (HR: 2.15), and kidney (HR: 2.17) diseases were independently associated with death.

**Conclusion:**

Our results prove that MPI predicts short-, mid-, and even long-term mortality in T2DM patients, whose death seems to be related to age and cognitive status, and even more to vascular and kidney diseases.

## Introduction

The increasing aging of the population has led to a proportional increase in the incidence of age-related conditions and diseases as well, such as sarcopenia [1], high falling risk, and reduced physical performance [2, 3], which lead to increased mortality, and also mild and severe neurocognitive disorders [4], metabolic syndrome [5], multifactorial anemia [6], and cardiovascular diseases, such as hypertension [7], cardiac failure [8], and atrial fibrillation [9]. Among them, Diabetes Mellitus (DM) deserves an honorable mention. It indicates a common metabolic disease, characterized by chronic hyperglycemia resulting from a deficit in insulin secretion and/or, most commonly, action [10–12]. The most prevalent form is called “type 2”, increasing when increasing age is called “type 2” (T2DM) [13], to differentiate it from “type 1”, caused by an absolute insulin secretion because of autoimmune mechanisms [14]. Elderly people are frequently affected by T2DM, often combined with other cardiometabolic disorders such as visceral obesity or hypertension [15–17]. It represents one of the most common diseases worldwide [11], and in Italy: the ARNO Diabetes Observatory showed an incidence rate of 5.83 per 1000 persons-years [18], and a prevalence of 6.2% [19], with growing incidence rates when growing age. Oral antidiabetic drugs [20–22], the prototype of which is metformin [23], increased survival rates and quality of life [24, 25]. Advanced forms of T2DM can instead require constant administration of insulin [26], which appeared to be over-prescribed in Italy, according to the ARNO Diabetes Observatory data [27]. Most recently, new drugs have been able to substantially change the history of the disease, namely sodium-glucose cotransporter 2 (SGLT2) inhibitors, and glucagon-like peptide-1 receptor agonists (GLP1-RA). SGLT2 inhibitors are associated with a reduction in body mass and pressure [28], improving cardiorenal outcomes [29]; GLP1-RA nowadays represents a second-line treatment in T2DM [30], and, as described for SGLT2 inhibitors, are associated with a reduction of cardiovascular risk, probably also thanks to crucial effects on renin-angiotensin system [31].

Being able to predict death assumes particular importance in elderly people, and comprehensive geriatric assessment (CGA) offers a specialistic help in this sense, by holistically examining elderly people, often with multiple diseases and impaired domains [32]. Pilotto A. et al., in 2008, validated the Multidimensional Prognostic Index (MPI) [33], a useful multidimensional tool which divides the patients into risk classes (low, moderate, and severe), predicting their probability of exitus, rehospitalization, and institutionalization. Starting life for the assessment of hospitalized subjects [33], it received subsequent validations in other settings, like the outpatient one [34, 35], intermediate care facilities [36], and, more recently, even for telephone administration [37]. It was also studied in specific populations, e.g., patients with myocardial infarction [38], acute respiratory failure [39], hip fracture [40, 41], chronic kidney disease [42], DM [43, 44], cancer [45, 46], and even COVID-19 [47]. About studies performed on DM, to the best of our knowledge, they evaluated the patients’ data for a maximum of 3 years after MPI administration [44]. Moreover, although based on common assumptions, that is the usual exclusion of older adults from trials, in the mentioned study [44], the authors specifically focused on statin treatment, demonstrating its association with reduced mortality in DM patients.

According to that, the aim of the present work is to study MPI accuracy in predicting mortality in a cohort of T2DM patients followed-up for 13 years.

## Methods

### Design of the study

This prospective study included subjects evaluated at the Geriatric Outpatient Service of the University Hospital of Monserrato, Cagliari, Italy, in 2009 and followed-up for a 13-year period.

### Inclusion criteria

Age ≥ 65 years; diagnosis of T2DM.

### Exclusion criteria

Age < 65 years; absence of T2DM diagnosis; contraindication to MPI (e.g., acute confusion, delirium), informed consent not provided.

### Assessment

The enrolled subjects were evaluated with MPI, which ranges from 0 to 1, identifying three levels of risk: MPI1 (low risk, 0.0–0.33), MPI2 (moderate risk, 0.34–0.66), and MPI3 (severe risk, 0.67–1.0) [33]. It is developed by considering the total scores of eight domains:

- Short Portable Mental Status Questionnaire (SPMSQ) [48], for cognitive assessment. It includes 10 items, and its total scores, corrected for years of school, ranges from 0 (absence of cognitive impairment) to 10 (maximum impairment). Scores < 5 indicate no or mild impairment, from 5 to 7 moderate impairment, and from 8 to 10 severe impairment
- Activities of Daily Living (ADL) [49], for the assessment of residual autonomies. Its total score ranges from 6 (complete independence) to 0 (complete dependence)
- Instrumental Activities of Daily Living (IADL) [49], for the assessment of residual autonomies. Its total score ranges from 8 (independence) to 0 (complete dependence)
- Mini Nutritional Assessment (MNA) [50, 51], for the assessment of nutritional status. Its total score ranges from 30 (excellent nutritional status) to 0 (severe malnutrition). Scores < 17 indicate malnutrition, 17–23.5 risk of malnutrition, ≥ 24 adequate nutritional status
- Exton-Smith Scale (ESS) [52], for the assessment of the risk of pressure injuries. Its total score ranges from 20 (absence of risk) to 5 (maximum risk). Scores ≤ 12 indicate a surely increased risk
- Comorbidity Index Rating Scale (CIRS) [53], for the assessment of the comorbidity burden. It evaluates 14 categories of pathologies concerning some organs and systems, hypertension, psychiatric and behavioral aspects. The Comorbidity Index (CIRS-IC) corresponds to the number of categories with ≥ 3 score, indicating an at least moderate and needing treatment alteration
- Number of different drugs taken
- Social support (household composition, institutionalization, services)

They were also evaluated with:

- Glycated hemoglobin (HbA1c) [54], for the estimation of long-term glycemic control
- Years since T2DM diagnosis

The abovementioned assessment was administered by trained geriatricians in the outpatient setting.

### Statistical analysis

Variables were expressed as means and SDs or in percentages (%), where appropriate. Kolmogorov–Smirnov method was used to test normal distribution in continuous variables. Chi-squared test (*χ*^2^) and Student’s *t*-test were used to compare the variables in the groups. Cox proportional hazard model was designed in order to estimate the survival probability: its results were expressed as Hazard Ratios (HRs), and *p* values > 0.1 were excluded from the model.

The results are reported indicating *p* values in reference to a 95% Confidence Interval (CI).

MedCalc software (Version 20.218, Ostend, Belgium) was used for the statistical analysis.

Considering confidence level: 95%, confidence interval: 5%, standard deviation (SD): 0.5, *Z*-score (*z*): 1.96, and error margin (*e*): 10%, the final sample (*N*) should consist of at least 96 subjects, according to the formula

$$N= \frac{{z}^{2}\times \mathrm{SD} (1-\mathrm{SD})}{{e}^{2}}$$

## Results

According to inclusion criteria, our study included 107 community-dwelling people aged 65 years or more, of whom 63 were women (58.9%), with an average (SD) age of 74.9 years (5.9). The characteristics of the sample are shown in Table 1.

**Table 1:** Characteristics of the sample

Variable	Minimum	Maximum	Mean	SD
Age (years)	65	89	74.9	5.9
MPI	0	0.69	0.2	0.2
SPMSQ	0	8	1.2	1.5
ADL	1	6	5.2	1.1
IADL	0	8	6.9	1.9
MNA	12	29.5	23.9	3.7
ESS	13	20	18.6	1.9
CIRS-IC	1	9	4.2	1.6
Drugs taken (number)	0	12	5.2	2.6
HbA1c (%)	4.6	14	7.0	1.5
Years since T2DM diagnosis	< 1	46	11.6	10.7

Variable	*n*.	%
MPI1	87	81.3
MPI2	17	15.9
MPI3	3	2.8
Living alone	21	19.6
Living in family (or with other support)	84	78.5
Living in institution	2	1.9

*SD* standard deviation, *MPI* Multidimensional Prognostic Index, *SPMSQ* Short Portable Mental State Questionnaire, *ADL* Activities of Daily Living, *IADL* Instrumental Activities of Daily Living, *MNA* Mini Nutritional Assessment, *ESS* Exton-Smith Scale, *CIRS-IC* Cumulative Illness Rating Scale-Comorbidity Index, *HbA1c* glycated hemoglobin, *T2DM* type 2 diabetes mellitus, *n.* number, *MPI* Multidimensional Prognostic Index, *MPI1* MPI 0.0–0.33 (low risk of adverse event), *MPI2* MPI 0.34–0.66 (moderate risk of adverse event), *MPI3* MPI 0.67–1.00 (severe risk of adverse event)

We divided the sample into three groups, according to MPI scores, obtaining MPI1 (0.0–0.33, made up of 87 subjects), MPI2 (0.34–0.66, made up of 17 subjects), and MPI3 (0.67–1.0, made up of 3 subjects). Since MPI3 presented a significantly lower number of subjects, it was excluded by further analysis. In Table 2, the more common comorbidities are summarized and divided into two groups. The most frequent was hypertension (83.6% of the sample). Peripheral vascular (33.3% vs 64.7%, *p* = 0.0155), ear/eye (49.4% vs 76.5%, *p* = 0.0417), musculoskeletal (29.9% vs 58.8%, *p* = 0.0224), and neurological diseases (12.6% vs 41.2%, *p* = 0.0046) were significantly more common in MPI2 than in MPI1. As in Table 3, 36.5% of the sample was receiving only diet therapy, while metformin was the most common oral antidiabetic drug taken (28.9%). Moreover, 31.7% took at least one type of insulin (“rapid” and/or “slow”): in particular, 20.6% took “rapid” insulin, 27.1% “slow” insulin, and 14% followed a multiple daily injection therapy. Finally, 7.5% of the patients followed a multidrug regimen, and 6.5% took basal-supported oral therapy (BOT). About other drugs, diuretics (28.7% vs 58.8%, *p* = 0.0169) and proton pump inhibitors (24.1 vs 52.9%, *p* = 0.0170) were more commonly taken by MPI2 than MPI1 patients.

**Table 2:** Comorbidities in MPI1 and MPI2 subgroups

Comorbidity	Percentage	MPI1	MPI2	*p*
		(*n*. 87)	(*n*. 17)	
		Percentage	Percentage	
Hypertension	83.6	81.6	94.1	0.2043
Cardiological disease	46.1	42.5	64.7	0.0950
Vascular disease	38.5	33.3	64.7	**0.0155**
Respiratory disease	13.5	13.8	11.8	0.8235
Ear or eye disease	54.8	49.4	76.5	**0.0417**
Upper gastrointestinal disease	18.3	16.1	29.4	0.1958
Lower gastrointestinal disease	12.5	12.6	11.8	0.9205
Liver disease	13.5	13.8	11.8	0.8235
Kidney disease	15.4	13.8	11.8	0.8235
Genitourinary disease	21.1	24.1	5.9	0.0934
Musculoskeletal disease	34.6	29.9	58.8	**0.0224**
Neurological disease	17.3	12.6	41.2	**0.0046**
Endocrine disease (except diabetes)	74.0	71.3	88.2	0.1463
Mood or cognitive disease	10.6	10.3	11.8	0.8624
Active or past neoplasia	10.6	10.3	11.8	0.8624
Immunological disease	6.7	4.6	17.6	0.0506

Bold indicates p < 0.05

*MPI* Multidimensional Prognostic Index, *MPI1* MPI 0.0–0.33 (low risk of adverse event), *MPI2* MPI 0.34–0.66 (moderate risk of adverse event)

**Table 3:** Drugs taken in MPI1 and MPI2 subgroups

Drug	Percentage	MPI1	MPI2	*p*
		(*n*. 87)	(*n*. 17)	
		Percentage	Percentage	
Diet therapy	36.5	39.1	23.5	0.2255
Oral Antidiabetic^a^	36.5	35.6	41.2	0.6657
Insulin^b^	31.7	29.9	47.1	0.1694
Beta-blocker	25.0	21.8	41.2	0.0937
Calcium channel blocker	29.8	29.9	29.4	0.9600
Diuretic	33.6	28.7	58.8	**0.0169**
ACE inhibitor	42.3	40.2	52.9	0.3343
Sartan	27.9	26.4	35.3	0.4585
Statin	46.1	47.1	41.2	0.6542
Antiplatelet	40.4	36.8	58.8	0.0918
Proton pump inhibitor	28.8	24.1	52.9	**0.0170**
Steroid	6.7	6.9	5.9	0.9793

Bold indicates p < 0.05

*MPI* Multidimensional Prognostic Index, *MPI1* MPI 0.0–0.33 (low risk of adverse event), *MPI2* MPI 0.34–0.66 (moderate risk of adverse event)

^a^Oral antidiabetic: metformin (28.9%), sulfonylureas (8.4%), glitazones (1.9%)

^b^Insulin: basal (glargine, detemir) (27.1%), bolus (lispro, aspart, glulisine) (20.6%)

As in Table 4, age, ADL, IADL, MNA, and ESS were significantly higher in MPI1 than in MPI2. On the contrary, SPMSQ, CIRS-CI, number of drugs taken, and years since T2DM diagnosis were significantly lower in MPI1 than MPI2. The analysis also showed that HbA1c values did not significantly differ among the groups.

**Table 4:** Anthropometric, psychometric and clinical differences in MPI1 and MPI2 subgroups

Variable	MPI1 (*n*. 87)		MPI2 (*n*. 17)		*p* value
	Mean	SD	Mean	SD	
Age (years)	73.8	5.7	79.4	4.2	**0.0002**
SPMSQ	0.9	0.9	2.1	1.7	**0.0001**
ADL	5.5	0.7	4.4	1.5	**< 0.0001**
IADL	7.5	1.1	5.1	2.61	**< 0.0001**
MNA	24.5	3.41	22.1	3.3	**0.0077**
ESS	19.1	1.3	16.8	2.1	**< 0.0001**
CIRS-IC	3.9	1.5	5.4	1.5	**< 0.0001**
Drugs taken (*n*.)	4.6	2.2	7.6	2.2	**< 0.0001**
HbA1c	6.9	1.4	7.7	1.9	0.0555
Years since T2DM diagnosis	9.8	9.0	17.8	13.3	**0.0026**

Bold indicates p < 0.05

*MPI* Multidimensional Prognostic Index, *MPI1* MPI 0.0–0.33 (low risk of adverse event), *MPI2* MPI 0.34–0.66 (moderate risk of adverse event), *SD* standard deviation, *MPI* Multidimensional Prognostic Index, *SPMSQ* Short Portable Mental State Questionnaire, *ADL* Activities of Daily Living, *IADL* Instrumental Activities of Daily Living, *MNA* Mini Nutritional Assessment, *ESS* Exton-Smith Scale, *CIRS-IC* Cumulative Illness Rating Scale-Comorbidity Index, *n.* number, *HbA1c* glycated hemoglobin, *T2DM* type 2 diabetes mellitus

**Fig. 1:**
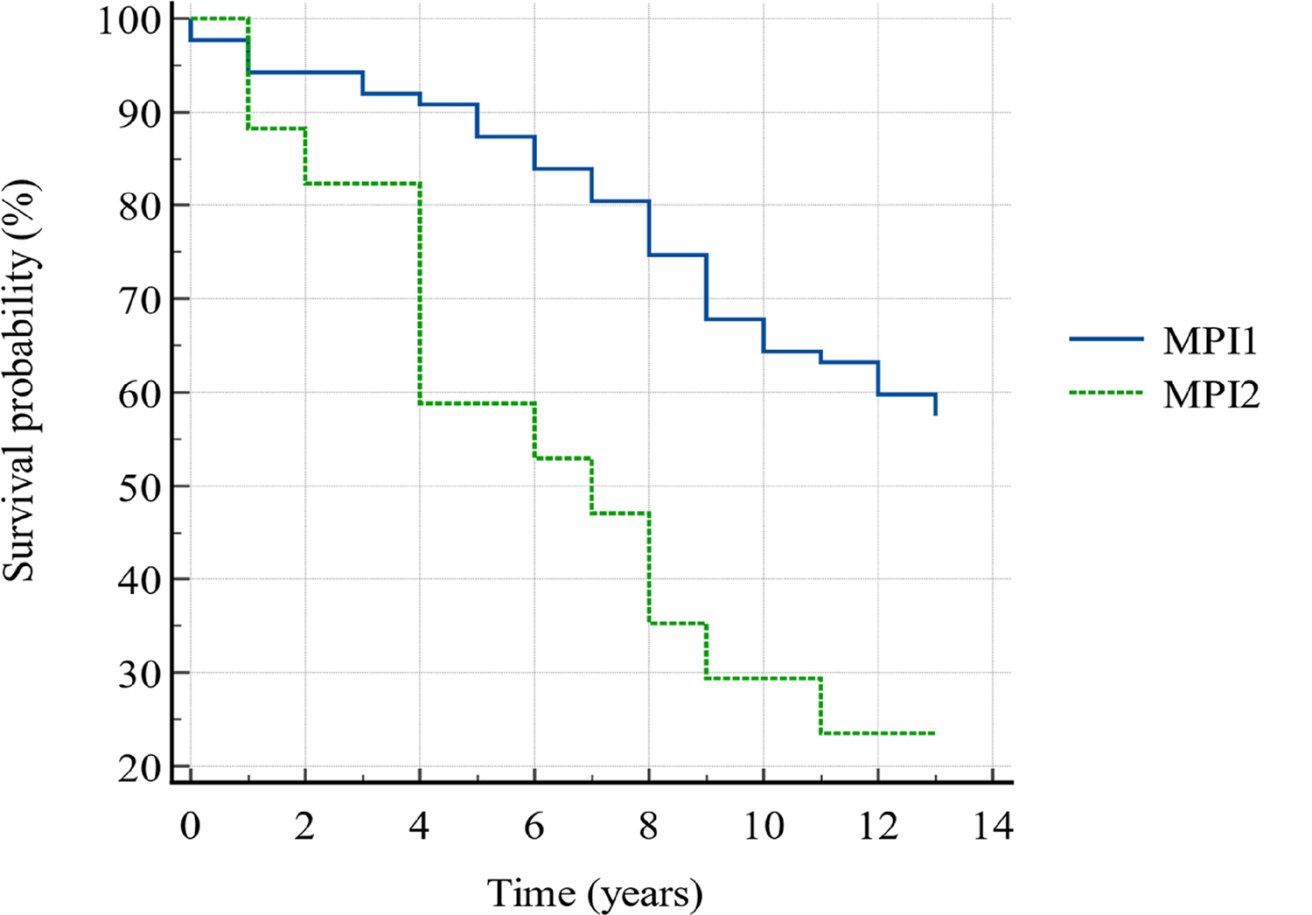
Survival curves. *MPI* Multidimensional Prognostic Index, *MPI1* MPI 0.0–0.33 (low risk of adverse event), *MPI2* MPI 0.34–0.66 (moderate risk of adverse event)

The whole sample was followed-up for 13 years, and the overall 13-year survival rate was 51.9% (the analysis of year-by-year-survival rates is shown in Table 5). The comparison of survival curves (Figure 1) revealed a significant difference among the two groups (*p* = 0.0007). MPI1 group presented higher survival rates than MPI2 since year 1 (94.3% vs 88.2%), afterwards the gap among them widening, until reaching 63.2% vs 23.5% survival rate on the eleventh year, as confirmed by the HR of 4.71 (CI 1.91–11.57). For the sake of completeness, the MPI3’s 2-years survival rate was 0%, and merging MPI2 and MPI3 the abovementioned gap widened to 63.2% vs 20.0% on the eleventh year (HR: 6.29, CI 2.68–14.79).

**Table 5:** Survival rates

Survival time (years)	MPI1		MPI2		Overall	
	Survival proportion	Standard error	Survival proportion	Standard error	Survival proportion	Standard error
< 1	0.977	0.0161	–	–	0.981	0.0135
1	0.943	0.0250	0.882	0.0781	0.933	0.0246
2	–	–	0.824	0.0925	0.923	0.0261
3	0.920	0.0292	–	–	0.904	0.0289
4	0.908	0.0310	0.588	0.119	0.856	0.0345
5	0.874	0.0356	–	–	0.827	0.0371
6	0.839	0.0394	0.529	0.121	0.788	0.0400
7	0.805	0.0425	0.471	0.121	0.750	0.0425
8	0.747	0.0466	0.353	0.116	0.683	0.0456
9	0.678	0.0501	0.294	0.111	0.615	0.0477
10	0.644	0.0513	–	–	0.587	0.0483
11	0.632	0.0517	0.235	0.103	0.567	0.0486
12	0.598	0.0526	–	–	0.538	0.0489
13	0.575	0.0530	–	–	0.519	0.0490

*MPI* Multidimensional Prognostic Index, *MPI1* MPI 0.0–0.33 (low risk of adverse event), *MPI2* MPI 0.34–0.66 (moderate risk of adverse event)

**Fig. 2:**
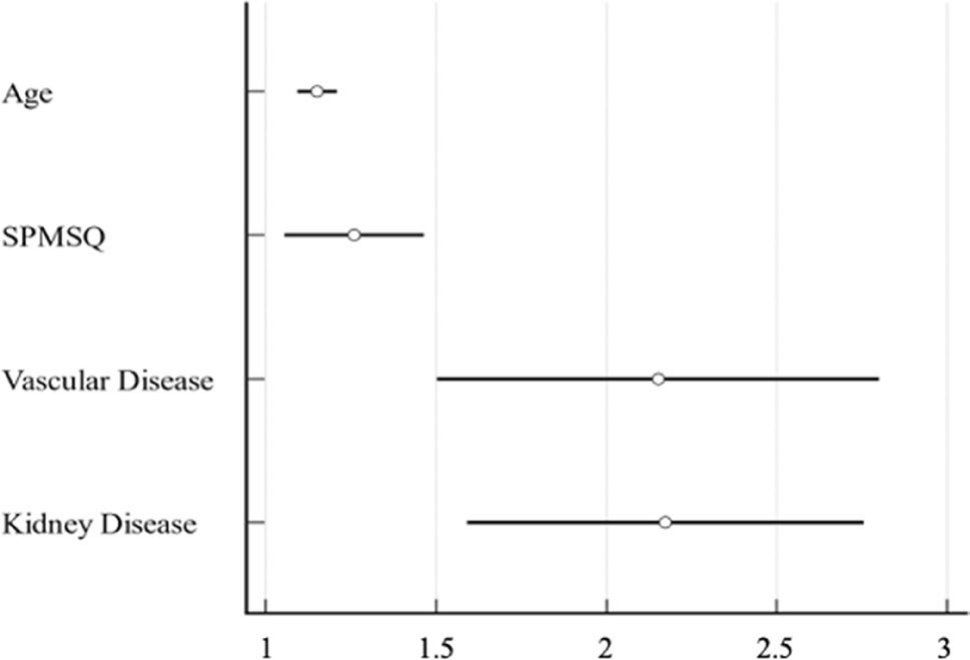
Covariates in survival. *SPMSQ* Short Portable Mental State Questionnaire

The Cox regression model, conducted in order to study the independence of different variables associated with the “death” outcome, included age, SPMSQ, ADL, IADL, MNA, CIRS-IC, numbers of drugs taken, HbA1c, years since T2DM diagnosis, and also the presence/absence of the diseases listed in Table 2, and the assumption of the drugs listed in Table 3. It highlighted age (HR: 1.15, CI 1.01–1.22, *p* < 0.0001), SPMSQ (HR: 1.26, CI 1.03–1.55, *p* = 0.0274), vascular (HR: 2.15, CI 1.25–4.12, *p* = 0.0205) and kidney (HR: 2.17, CI 1.21–3.89, *p* = 0.0089) diseases to be significantly associated with the event, while the others were excluded by the model, as in Figure 2. In particular, among vascular comorbidities, carotid atherosclerosis (42.5%), and chronic arteriopathy of the lower limbs (27.5%) were the most frequent in the sample, while cystic kidney disease (62.5%), and chronic kidney disease (31.25%) were the most represented among kidney comorbidities.

## Discussion

Nowadays, T2DM represents one of the most common metabolic diseases worldwide, especially in the elderly [11, 13, 18, 19]. CGA is a specialistic tool to holistically assess older adults [32], and MPI represents one of its expressions [33] in various settings and for several diseases. Nevertheless, to the best of our knowledge, no previous focused-on-DM MPI application considered longer than 3 years outcomes. Our work fits into this line of research, aiming to study, in a cohort of T2DM patients, MPI accuracy not only in predicting short- and mid- [43, 44], but even long-term mortality. In order to achieve such an objective, we recruited subjects aged 65 years or more, with an average age of 74.9 years and followed them up for 13 years.

In the study population, the average risk of the negative event was low, cognitive abilities were adequate, and so were the autonomies in performing basic and instrumental activities of daily living as well; the population also presented a risk of malnutrition, mild risk of pressure injuries, and polypharmacotherapy [55], an important issue in elderly given the high number of under- and over-prescriptions in several diseases [56, 57]. Moreover, it averagely presented more than 4 needing treatment conditions. As far as it concerns average (SD) glycated hemoglobin, it was 7 (1.5), representing satisfactory glycemic control for elderly diabetics [53, 58].

The variables were studied among two out of three groups based on risk stratification (MPI1, low, and MPI2, moderate risk, while MPI3, severe risk of an adverse event, made up of a significantly lower number than the others, was excluded by the analysis). Anyway, overall, cognitive performances, autonomies in daily living, nutritional status, risk of pressure injuries, comorbidities, and taken drugs were better (*p* ≤ 0.0077) in people with lower risk. It goes without saying that such an association is coherent with MPI calculation – being it built according to the abovementioned variables [33], while an interesting data that emerged was that people with the low risk presented a shorter story of DM than people with moderate and high risk as well (*p* = 0.0026). Another significant result is represented by the fact that glycated hemoglobin percentage did not show a significant difference (*p* = 0.0555) among the groups. It would be explained by the fact that the two groups largely differed in size: the worse glycemic control might possibly affect the increased mortality in the MPI2 group.

Following the aim of the study, we considered the deaths over 13 years, obtaining survival curves: they showed that the MPI2 group presented more than 470% of risk to die (HR: 4.71) with respect to MPI1. This deeply increased mortality cannot depend only on the higher age in MPI2 patients, but it is rather certainly influenced also by the abovementioned more compromised general status of the group, and possibly the worse glycemic control. These aspects were reinforced by the fact that multivariate analysis showed that increased age (HR: 1.15), poorer cognitive abilities (HR: 1.26), and the presence of vascular and kidney diseases (HRs: 2.15 and 2.17, respectively) were independently associated with long-term death. What emerged is consistent with the literature [59–61], and even with the natural history of diabetes, the most common complications of which are really represented by vascular and kidney affections [62].

Unfortunately, being MPI3 made up of a too low number of subjects, the HRs concerning it would not have been statistically significant, though they would have likely shown a clear tendency, owing to the fact that the whole group had passed away by the second follow-up year.

This is the first study monitoring DM patients evaluated with MPI over such a long period, and this represents its greatest strength. Obviously, we recognize some limitations, represented by the monocentric nature of the study, and the relatively low number of enrolled subjects, especially for the MPI3 group, all the more so because we believe that a higher representation of this group could have further reinforced the results. Another limitation is represented by the absence of data related to new drugs (SGLT2 inhibitors, GLP1-RA), which as previously stated [28–31] have modified the history of DM and the prevalence of its complications.

## Conclusions

In conclusion, this study proved that MPI is able to predict short-, mid- (as previously demonstrated [44]), and even long-term mortality in T2DM patients, whose death seems to be related to age, cognitive status, and vascular and kidney diseases. Further studies with larger samples and longitudinal follow-up are needed to confirm and deepen our results, especially since new antidiabetic agents have proved to influence cardiovascular and all-cause mortality in DM.

## Data Availability

The data and materials used and/or analyzed during the current study are not publicly available. They are available from the corresponding author upon reasonable request.

## Abbreviations

ADL: Activities of daily living
CI: 95% Confidence interval
CIRS: Cumulative illness rating scale
CIRS-IC: Cumulative illness rating scale-comorbidity index
CGA: Comprehensive geriatric assessment
DM: Diabetes mellitus
ESS: Exton-Smith scale
HbA1c: Glycated hemoglobin
IADL: Instrumental activities of daily living
MNA: Mini nutritional assessment
MPI: Multidimensional prognostic index
MPI1: MPI 0.0–0.33
MPI2: MPI 0.34–0.66
MPI3: MPI 0.67–1.00
*n*.: Number
SD: Standard deviation
SPMSQ: Short Portable Mental State Questionnaire
T2DM: Type 2 diabetes mellitus
